# 1832. Rise in Deaths from Firearm Injury and Drug Overdose During COVID-19 Pandemic and its Impact on Organ Donation in the United States

**DOI:** 10.1093/ofid/ofad500.1661

**Published:** 2023-11-27

**Authors:** Roshan Dhand, Abhay Dhand, Seigo Nishida, Kenji Okumura

**Affiliations:** Horace Greeley High School, Chappaqua, NY, Chappaqua, New York; Westchester Medical Center, Valhalla, NY, Valhalla, NY; Westchester Medical Center, Valhalla, NY, Valhalla, NY; Westchester Medical Center, Valhalla, NY, Valhalla, NY

## Abstract

**Background:**

COVID-19 pandemic had profound societal impact in United States (US) with decrease in overall life expectancy and associated increase in polysubstance abuse.

**Methods:**

Crude rates of population-based deaths among adults (18-75 years) from firearm injury and drug overdose were obtained from centers of disease control (CDC) WONDER database from period of 2011- 2021. Crude rates of underlying causes of donor (18-75 years) deaths from 2011- 2021 were obtained from United Network for Organ Sharing (UNOS) database. Trends of cause of death and impact of COVID-19 period on deaths from drug overdose and firearm injury were analyzed using joint point regression analysis. Correlation between population and organ-donor deaths was analyzed using spearman's rank correlation coefficient.

**Results:**

Average annual percentage change (AAPC) in deaths among the US population from drug overdose was 9.5 % and from firearm injury was 3.4%, with the highest ever rates from both causes in the US seen in 2021 (figure 1).

AAPC in cause of death among donors of organs for transplantation was 10.9% from drug overdose and 2.1% from firearm injury (figure 2).

There was a significant progressive increase in deaths from drug overdose (1.48 fold) and firearm injury (1.2 fold) from 2019 to 2021 (P < 0.01).

There was a significant correlation between population and organ-donor deaths from drug overdose and firearm injury during the COVID-19 period of 2020-2021 ( P < 0.01).

**Figure:**
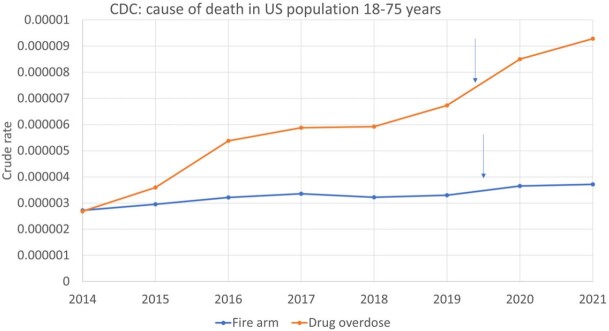

rates per 100,000 population

**Figure:**
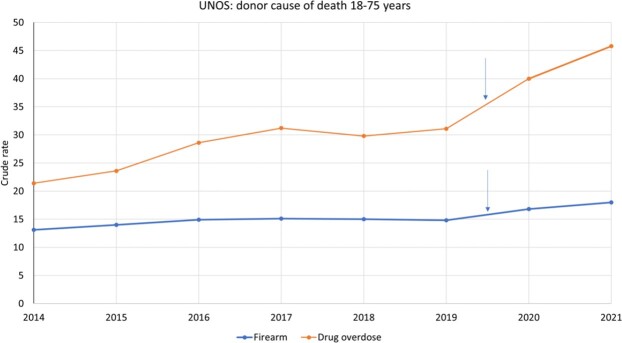

rates per 100,000 population

**Conclusion:**

COVID-19 pandemic has further exacerbated the trends in US population of deaths from drug overdose and firearm injury (including suicide and homicide). There was a consequent increase in organ donation for transplantation from donors who died from these two causes. Identifying socio-economic, regional and racial discrepancies associated with this rise can help guide public health policies for the immediate and long-term future. Unique risk factors, including increased infectious risk, associated with use of organs from these donors and subsequent long-term graft outcomes in the recipients will need to be monitored.

**Disclosures:**

**All Authors**: No reported disclosures

